# The development and theoretical application of an implementation framework for dialectical behaviour therapy: a critical literature review

**DOI:** 10.1186/s40479-019-0102-7

**Published:** 2019-02-12

**Authors:** Gill Toms, Lynne Williams, Jo Rycroft-Malone, Michaela Swales, Janet Feigenbaum

**Affiliations:** 1 Gill Toms, School of Healthcare Sciences, Bangor University, Fron Heulog, Bangor, Gwynedd LL57 2EF UK; 2 North Wales Clinical Psychology Programme, School of Psychology, Brigantia Building, Bangor University, Bangor, Gwynedd LL57 2DG UK; 3 Research Department of Clinical, Education and Health Psychology, University College London, Gower Street, London, WC1E 6BT UK

**Keywords:** Dialectical behaviour therapy, Implementation, Psychological therapy, Review

## Abstract

**Background:**

Dialectical behaviour therapy (DBT) is a third wave behaviour therapy combining behaviour based components with elements of mindfulness. Although DBT effectiveness has been explored, relatively little attention has been given to its implementation. Frameworks are often the basis for gathering information about implementation and can also direct how the implementation of an intervention is conducted. Using existing implementation frameworks, this critical literature review scoped the DBT implementation literature to develop and refine a bespoke DBT implementation framework.

**Method and results:**

The initial framework was developed by consolidating existing implementation frameworks and published guidance on DBT implementation. The critical literature review retrieved papers from Medline, CINAHL, PsycInfo, PubMed, and the reference lists of included papers. Framework elements were used as codes which were applied to the literature and guided the synthesis. Findings from the synthesis refined the framework.

The critical literature review retrieved 60 papers but only 14 of these explicitly focused on implementation. The DBT implementation framework captured all the execution barriers and facilitators described in the literature. However, the evidence synthesis led to a more parsimonious framework as some elements (e.g., research and published guidance) were seldom discussed in DBT implementation.

**Conclusion:**

To our knowledge this is the first published review exploring DBT implementation. The literature synthesis suggests some tentative recommendations which warrant further exploration. For instance, if DBT implementation is not pre-planned, having someone in the organisation who champions DBT can be advantageous. However, as the literature is limited and has methodological limitations, further prospective studies of DBT implementation are needed.

**Electronic supplementary material:**

The online version of this article (10.1186/s40479-019-0102-7) contains supplementary material, which is available to authorized users.

## Background

Dialectical Behaviour Therapy (DBT) [1] synthesises behavioural based therapy components (orientated towards change) with elements from mindfulness (orientated towards increasing acceptance). DBT is typically offered to people with a diagnosis of Borderline Personality Disorder (BPD) and a history of suicidal and self-harming behaviour. Therapists aim to impart new skills and develop clients’ behavioural flexibility to draw on appropriate skills in any given social or emotional situation. Core treatment components include individual therapy, telephone skills coaching, skills group and a clinician consultation team (where DBT therapists access support and guidance from other DBT team members), although services may only deliver some of these components (e.g. [2]). Several reviews summarising the evidence for DBT effectiveness are available (e.g. [3, 4]).

Implementation is the process through which the uptake of evidence-based interventions in routine clinical practice is systematically promoted. Transdisciplinary implementation frameworks exist, for instance, Promoting Action on Research Implementation in Health Services (PARIHS: [5, 6]), the Consolidated Framework for implementation for implementation research (CFIR: [7]), and the Core Implementation Components model ([8, 9]). The PARIHS framework covers many of the core elements of these models: context, evidence, facilitation and intervention elements. Context refers to the environment or setting that the implementation takes place in. Evidence can be derived from research, clinical experience or patient preference. Facilitation refers to the people and processes that support implementation and the intervention element demotes the characteristics of the intervention to be implemented. CFIR has an additional element related to implementation processes, which describes the practical implementation tasks undertaken. Each of these elements are sub-divided. For instance, PARIHS sub-divides evidence into research and published guidance, clinical experience and professional knowledge, preferences and experiences, and local knowledge. National implementations refer to many of these elements in their guidance, for example, the Increasing Access to Psychological Therapies manual [10].

DBT has unique features, such as, a multicomponent therapy process, telephone skills coaching, and a consultation team. The characteristics of people with BPD (the core client group) could also necessitate bespoke implementation strategies. The question of how best to implement a DBT intervention arose in the context of the Enabling and Motivating people (with a Personality Disorder) in Occupation, Wellbeing, Education and Responsibility (EMPOWER) research programme (NIHR Programme Grant: RP-PG-1212-20,011), which is developing and evaluating a DBT- Skills for Employment (DBT-SE) intervention. The evidence about DBT implementation has seldom been reviewed and this work was undertaken with a view to developing an implementation toolkit for the DBT-SE intervention. We aimed to review the DBT implementation literature to develop and refine a bespoke DBT implementation framework.

## Methods

### Framework development

**Fig. 1:**
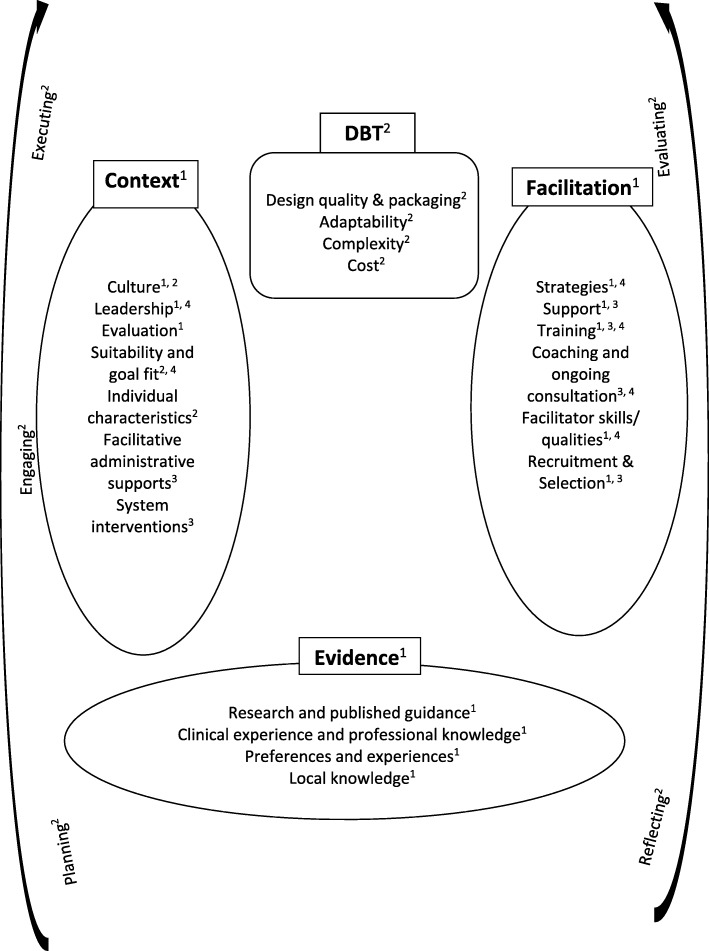
DBT implementation framework: first iteration. Key: PARIHS, 2004^1^; Damschroder et al., 2009^2^; Fixsen & Blasé, 2009^3^, Swales 2010a, 2010b^4^

To create an initial DBT implementation framework, elements from the main transdisciplinary frameworks (PARIHS, CFIR, and the Core Implementation Components model) and from published DBT implementation guidance [11, 12] were synthesised. PARIHS [5, 6] was selected as the underpinning framework as it highlights the pivotal role of contextual factors. Although it developed from existing models, this is the first framework to incorporate implementation insights from the DBT literature. The initial framework is represented in Fig. 1 and is described in Additional file 1.

### Critical literature review

To refine the framework, a critical literature review [13] was conducted. In critical reviews, the synthesis process is used to create a new model or a model embodying existing theory which then provides a ‘launch pad’ for subsequent testing. One of the strengths of this type of review lies in the analysis undertaken to create the model [13].

Four databases were searched with the terms ‘DBT’ and ‘Implementation’ in January 2016; Medline (EBSCO), CINAHL (EBSCO), PsycInfo (ProQuest), and PubMed (NCBI). These databases were selected as they hold health and psychology related literature. Reference lists of included papers were additionally screened as implementation issues might be discussed without this term being used as a key word or included in the abstract or title. DBT was defined as any combination of components or interventions which were identified as DBT by the study authors (Additional file 2 contains an example search). Implementation was defined as the process of introducing and sustaining DBT in routine practice. All retrievals were managed in RefWorks, an online bibliographic management programme. Only peer-reviewed papers were included but no date or evidence type restrictions were applied. For resource and time reasons, only papers published in English were included. The first author conducted the review and the eligibility of database retrieved papers was checked by a second reviewer (reviewer agreement was 97% with all disagreements resolved through discussion).

Consistent with the critical review approach, papers were not excluded for methodological reasons [13]. However, prospective and retrospective studies of implementation were considered to provide the strongest evidence due to their explicit focus on implementation. Discussion pieces were judged to form the weakest evidence as the experiences they are based on are often not accessible for review. The data extracted from papers included; the design, context, methodology, implementation barriers and facilitators, as well as author conclusions and recommendations (Additional file 3 contains the data extraction template). Extracted data were discussed by the review team and where necessary the text was re-reviewed.

#### Evidence synthesis

Extracted data relating to implementation barriers and facilitators, conclusions and recommendations were coded using deductive content analysis. This process used the elements and sub-elements in the DBT implementation framework as code labels and assigned them to the data segments. Where the extracted data did not fit any existing codes, a new code name was added and this process continued until all the data were categorised. Coding was conducted by the first author and a second reviewer checked the coding applied to sixteen papers (10 % of the papers coded): although conservatively judged agreement was 66% (a criterion that the same sub-elements were coded in each paper), differences in coding were negligible and easily resolved through discussion. For instance, the most common cause for disagreement was which code best captured the data. The team reviewed the final synthesis to ensure it presented an accurate reflection of the data.

## Results

### Critical literature review

**Fig. 2:**
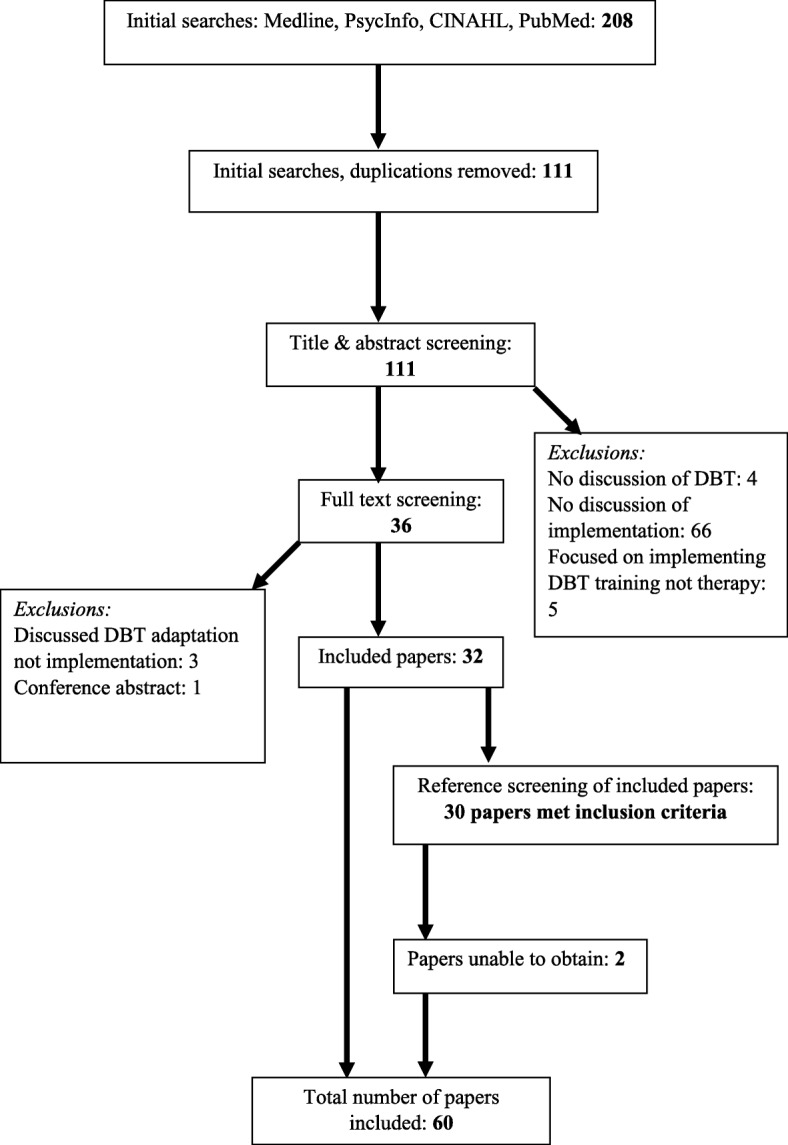
Literature review flow chart

Sixty-two papers met the inclusion criteria (32 from database and 30 from reference list searches), although two papers were unobtainable within the time limit of the review. The main reasons for exclusion were failure to consider DBT or a failure to discuss implementation issues (see Fig. 2). There were 11 discussion papers ([11, 12, 14–22]) and as these were considered the weakest form of evidence they were not included in the synthesis, but are detailed in Table 1. As seen in Table 2, nine papers collected retrospective ([23–31]) and five papers ([32–36]) collected prospective implementation data. There were 16 programme descriptions ([37–52]) and 19 trial process analyses ([53–71]). The majority (*N* = 38) of papers were from the United States (US), and most implementations of DBT were in mental health services.

**Table 1:** Discussion papers

Reference	Country & service context	Key points/ recommendations made
Chugani, 2015 [16]	America. College counselling centres	-Important to collect service-relevant outcome data as DBT is often adapted to fit the service -Important to adapt DBT so appropriate for the service -Can mitigate costs by hosting training or offering partial programmes
Borroughs & Somerville, 2013 [15]	America. Assertive Community Treatment teams	-There may be resource and financial barriers, especially in the US healthcare system where services cannot recoup costs for training, consultation team meetings or data collection -It is important to determine if DBT ‘fits’ the service’s client group and theoretical stance -Recommended adapting DBT and offsetting costs by using existing infrastructure and demonstrating cost-effectiveness
Koener, 2013 [18]	N/A	-DBT clinicians need a good conceptualisation of the therapy, including the treatment hierarchy and biosocial theory -Important that therapists are dialectical, cognitively flexible and validating -Recursive culture important; a community of therapists working with a community of patients, with everyone in the same boat -Services need to see patients as motivated to change and that services want to improve patient capability -Therapists should access the consultation team and mindfulness practice -Ensuring fidelity to manualised DBT ignores the contextual factors that moderate success
McHugh & Barlow, 2010 [19]	Worldwide; Reviews and describes a range of implementation efforts	-In America, Behavior Tech acts as a champion for DBT --Ongoing outcome monitoring important to sustain fidelity and quality improvement -Implementation issues have informed DBT training. For instance, teams implement DBT before completing final training so that they can access consultation after their first attempts
Swales, 2010a [11]	UK	-Larger DBT teams with less time will be slower at learning DBT than smaller teams who have greater allocated time -Important to gain staff commitment to implement DBT and to select staff with knowledge about DBT and implementation, who are willing to apply DBT skills themselves -Beneficial to recruit so that DBT teams encompass a range of skills -Important to have a DBT ‘champion’ and the team leader should be in a senior position -Consultation teams have an important role and the consultation agreement establishes the team climate -A minimum of two hours per work is necessary for supervision and consultation team meetings
Swales, 2010b [12]	UK	-Description of an organisational pre-treatment approach where the DBT team leader or champion: -Identifies the appropriateness of DBT, weighing the evidence, policy aims and organisation suitability, culture and climate -Considers the organisations experience in implementing other new therapies -Resolves competing goals and if synthesis is impossible undertakes a pros and cons analysis -Forms an advisory or steering group to address factors likely to interfere with implementation
Berzins & Trestman, 2004 [14]	America. Prison/correctional services. Non-systematic review and information collected from services	-All the programmes described had adapted DBT. There is currently no manual for DBT in correctional settings -Programmes were driven by clinical need (DBT had ‘goal fit’) -To implement and evaluate a proposed modified DBT programme for correctional settings, a coalition had been formed between the university, state and health departments
Huffman et al., 2003 [17]	N/A	-Champion/consultant should be willing to model DBT skills -To accommodate time limitations, single components of DBT can be applied rather than the comprehensive intervention -Need to provide psychoeducation about BPD and validate staff experience of difficulties -Use contingency management; frame behaviour modification as the most effective approach
Swenson et al., 2002 [21]	America. Public mental health authorities. Recommendations based on observations, a survey and literature review	-Barriers listed included therapist view of DBT suitability and staff turnover. Discussed therapist selection issues -Also discussed the barriers patients may face when starting DBT- e.g. it is a high time commitment and they might need to terminate current treatment contracts -Facilitators endorsed leadership from public mental health authorities, training, a positive attitude towards BPD and monitoring outcomes -Recommended forming coalitions between organisations providing DBT and those planning to implement DBT -Recommended providing training (psychoeducation) for public mental health authorities about DBT and BPD -Recommended highlighting to patients that DBT participation is voluntary
Scheel, 2000 [20]	N/A. Overview and literature critique	-Suggested inpatient settings might transition most easily to DBT, as there is fit in terms of time availability and goals -Need access to training, supervision and consultation -Implementing DBT in a manner consistent with the evidence base requires a considerable staff team: resources may threaten viability -Outpatient DBT requires inter-agency support (a need for coalitions)
Swenson, 2000 [22]	America	-Should use DBT skills to help implementation -The design of DBT contributes to its appeal to therapists. For instance, it integrates different orientations meaning it has a wide support base and therapists from various orientations automatically have ‘buy-in’ -DBT can be both pragmatic and very sophisticated

**Table 2:** Implementation papers, programme descriptions and trial process analysis papers

Reference	Country & service context	Paper type	Methodology	DBT outcomes	Implementation relevant outcomes
*Implementation papers*					
Chwalek & McKinney, 2015 [24]	America (and Germany). Range of mental health services	Retrospective data collection	Survey and interviews of music therapists	N/A	38.3% of respondents endorsed implementing DBT in music therapy practice
Ditty et al., 2015 [26]	America. Mental health services	Retrospective data collection	Survey and interviews with therapists trained in DBT exploring inner setting constructs of CFIR framework	N/A	96% of respondents provided individual therapy, 99% provided skills groups, 97% attended a consultation team and 87% provided phone skills coaching
Carmel et al., 2014 [23]	America. Public behaviour health system	Retrospective data collection	Telephone interviews with therapists	N/A	Therapists received ten days (80 h) of DBT training over 13 months
Herschell et al., 2014 [35]	America	Prospective data collection	Quantitative survey of therapists pre and post implementation	Therapists reported trend reduction in patient A&E visits and hospitalisations	Therapist training ranged from 32 to 96 h (maximum 96 h) and received on average 25.67 h of phone consultation
Swales et al., 2012 [31]	UK. Range of inpatient, outpatient and forensic services	Retrospective data collection	Telephone interviews with DBT team members	7.1% said improved patient outcomes were an implementation facilitator	62.8% of programmes remained active at five years. 57% of programmes provided all DBT components
Dimeff et al., 2011 [32]	America.	Prospective data collection	Randomised controlled trial with DBT naïve therapists	N/A	E-learning resulted in best knowledge retention at 15 week follow-up
Dimeff et al., 2009 [33]	America.	Prospective data collection	Randomised controlled trial with DBT naïve therapists	N/A	80% of therapists completed training. Online training best at improving knowledge. Instructor led training better than reading the training manual at increasing self-efficacy and satisfaction
Herschell et al., 2009 [36]	America. Community mental health services	Prospective data collection	Qualitative interviews pre implementation with county level mental health administrators	N/A	N/A
Perseuis et al., 2007 [28]	Sweden. Outpatient services	Retrospective data collection	Survey and interviews with DBT trained therapists	N/A	Therapists worked part-time in the DBT team. Tendency for greater staff burnout over time, but not statistically significant. Reduced occupational stress
Sharma et al., 2007 [30]	America. Psychiatric residency	Retrospective data collection	Survey of residency directors and senior residents. Also presented a case study	Patient hospitalised then discontinued DBT therapy	56% of residency programmes had no lectures on DBT and 32% provided no DBT supervision
Frederick & Comtois, 2006 [27]	America	Retrospective data collection	Survey of psychiatry residency graduates who had attended at least one DBT workshop	N/A	23% of respondents practiced all DBT components. Most practiced at least one DBT component
Cunningham et al., 2004 [25]	America	Retrospective data collection	Interviews with BPD patients who had received DBT therapy	Reduced hospitalisations and increased vocational work	N/A
Perseius et al., 2003 [29]	Sweden	Retrospective data collection	Interviews with DBT therapists and patients	Patients reported positive outcomes. Patients had been in therapy for at least 12 months	Therapists gained a new perspective and DBT influenced how therapists solved problems in their own lives
Hawkins & Sinha, 1998 [34]	America. Department of mental health and addiction services	Prospective data collection	Correlated therapist DBT knowledge to demographics and training through repeated measures and naturalistic service outcome data	Archival data suggested DBT training led to better patient outcomes: less A&E, inpatient, seclusion and restraint use	Training and the amount of time practiced DBT had a moderate correlation with DBT knowledge
*Other papers*					
James et al., 2015 [60]	America. Psychiatric facility	Trial process analysis	Service embedded repeated measures evaluation	Good outcomes	Grant funded participants had higher attrition
Kinsey & Reed, 2015 [43]	America. Native American tribe outpatient mental health and substance use service	Programme description	N/A	N/A	Programme had run for 14 years and had a good relationship with the tribal community
Baillie & Slater, 2014 [39]	UK. Community intellectual disability service	Programme description	Mostly discussion	Some evidence that patients developed emotion regulation and distress tolerance skills	DBT service had been in operation for four years
Engle et al., 2013 [42]	America. College counselling service	Programme description	Between groups	Reduced psychiatric and substance use hospitalisations. Reduced college absence due to mental health problems	Team received intensive training. Carried caseloads of up to seven patients plus one skills group
Arroyo et al., 2012 [38]	America. Mount Sinai East Harlem health outreach project	Programme description	N/A	Anecdotal evidence of patient improvement	Implemented skills group only. Therapists received fortnightly supervision
Lajoie et al., 2011 [44]	America. Residency run clinic	Programme description	N/A	N/A	Implemented all core DBT components
Morrissey & Ingamells, 2011 [47]	UK. Learning disability forensic secure service	Programme description	Naturalistic outcomes reported	Reduced symptoms and distress. Reduced perceived risk	Implemented programme over six years
Pasieczny & Connor, 2011 [66]	Australia. Adolescent mental health service	Trial process analysis	Between groups	Patients of intensively trained therapists had better outcomes in terms of DSH and suicide attempts	Therapists worked in DBT team part-time. Therapist adherence ranged nine-to-12 (maximum achievable = 12)
Little et al., 2010 [46]	America. Residential service	Programme description	N/A	Self-reported patient improvement and positive feedback	DBT was the best implemented treatment in the service; had furthest reach, most staff support and needed less senior administrative support. Minimal attrition
Sampl et al., 2010 [48]	America. Correctional setting	Programme description	N/A	N/A	Primarily just implemented skills group
Blennerhassett et al., 2009 [54]	Ireland. Community mental health team	Trial process paper	Repeated measures	Improved risks, symptoms, functioning and subjective wellbeing. Reduced hospitalisations and reduced costs	Therapists completed intensive training but DBT team not established in the service
Kerr et al., 2009 [62]	America. Low resourced rural training clinic	Trial process analysis	Case study	There were “meaningful” changes in suicidality and misery ratings	The therapist received DBT training and supervision. Could not access DBT skills group, so provided skills training in individual therapy sessions. Also provided adapted phone skills coaching
Hjalmarsson et al., 2008 [59]	Sweden. Outpatient services	Trial process analysis	Repeated measures	Patients had reduced para-suicidal behaviours and psychological distress	18 therapists trained and worked part-time on DBT team. DBT now provided by the service as a routine treatment. Attrition low
Woodberry & Popenoe, 2008 [71]	America. Adolescent and family outpatient clinic	Trial process analysis	Repeated measures	Good outcomes reported	Five therapists received intensive training, the rest received less intensive or in-service training. The hospital provided some money to support staff training
Comtois et al., 2007 [57]	America. Harbour view mental health services- community mental health centre	Trial process analysis	Repeated measures	Reduced DSH, A&E visits and inpatient admissions	Noted DBT staff were highly trained. Implemented all DBT components and incorporated access to DBT relevant services
Prendergast & McCausland, 2007 [67]	Australia, Adult mental health outpatient service	Trial process analysis	Between groups	Reduced depression and frequency of suicide attempts and hospitalisations. Improved patient functioning and reduced intervention duration	The team comprised 12 therapists. Attrition was 31%
Zinkler et al., 2007 [52]	UK. Newham project for BPD	Programme description	N/A	Reduced hospitalisation and DSH frequency	Annual service cost £92,000. Therapists worked part-time on DBT team. Staff satisfaction and retention high
Brassington & Krawitz, 2006 [56]	New Zealand. Mental health service	Pilot trial process analysis	Repeated measures	Good outcomes reported	Implementation reportedly successful. Team staffed by part-time therapists and at the end of the trial team had a dedicated budget
Koons et al., 2006 [65]	America. Division of vocational rehabilitation	Trial process analysis	Repeated measures	At six months improved depression, hopelessness, anger expression, work role satisfaction and number of hours worked	Provided just DBT skills group
Lew et al., 2006 [45]	America. Intellectual disability service	Programme description	Provided service outcome data	Eight learning disability patients completed the programme. DSH gradually reduced	Staff carried caseloads of eight. Parents and staff also attended the skills groups
Nelson-Gray et al., 2006 [64]	America. Outpatient adolescent clinic	Trial process analysis	Repeated measures	Reduced negative behaviours, externalising and internalising symptoms, and depression. Increased positive behaviours	Trained a high number of graduate students and these students achieved 88% intervention delivery fidelity over eight groups
Vitacco & Van Rybroek, 2006 [50]	America. Forensic hospitals	Programme description	Primarily a discussion paper	N/A	N/A
Nee & Farman, 2005 [63]	UK. Female prisons	Trial process analysis	Between groups (with a waiting list control)	The majority of completers showed overall improvement with notable effect sizes	Implementation problems believed to contribute to high attrition
APA Gold Award, 2004 [37]	America. Grove street adolescence residence- residential care service	Programme description	N/A	Outcome data indicated the programme was effective	Provided all DBT components and had 18.7 full time equivalent staff members
Ben-Porath et al., 2004 [53]	America. Urban community mental health centre	Trial process analysis	Repeated measures	Reduced life threatening, therapy interfering and QOL interfering behaviours	Implemented all core DBT components. Three of the eight DBT team members left within six months
Katz et al., 2004 [61]	Canada. Adolescent inpatient service	Pilot trial process analysis	Between groups	Reduced behavioural incidents on ward. Equivalent to TAU in reducing para-suicidal behaviour, depression symptoms and suicidal ideation at one year follow-up	Provided skills group, individual therapy and milieu therapy
Sunseri, 2004 [49]	America. Residential centre for adolescents	Programme description	Naturalistic outcomes reported	Reduced attrition, inpatient days and duration of restraint and seclusion	Staff confidence grew with DBT implementation
Eccleston & Sobello, 2002 [58]	Australia. Prison service	Pilot trial process analysis	Repeated measures	Trend improvement supported by patient feedback	Anecdotally, a range of staff saw programme benefits
Rathus & Miller, 2002 [68]	America. Adolescent outpatient clinic	Trial process analysis	Between groups	Reduced hospitalisations and increased retention but did not reduce suicide attempts	DBT transportable to real-world settings: provided in a hospital, not a university-based clinic
Trupin et al., 2002 [69]	America. Incarceration centre for female juvenile offenders	Trial process analysis	Between groups	Only one unit showed reduced behaviour problems	Only one unit showed less staff use of punitive responses. Not all staff adherent to DBT
van den Bosch et al., 2002 [70]	Netherlands. Addiction treatment centre	Trial process analysis	Randomised controlled trial	Reduced DSH but did not improve substance use	Over time therapists said they felt less isolated, more competent and experienced more work satisfaction. Consultation team attendance 100%. Attrition 37%
Bohus et al., 2000 [55]	Germany. Inpatient service	Pilot trial process analysis	Repeated measures	Reduced DSH, disassociation phenomena and depressive symptoms	Intervention was rated positively by staff and patients and this was an impetus to conduct the trial
Wolpow et al., 2000 [51]	America. Residential programme	Programme description	Included a service evaluation	Patients gave positive feedback and observations were positive	Residential staff became more positive about DBT
Gold Award, 1998 [41]	America. Mental health centre	Programme description	N/A	Positive patient outcomes and reduced costs reported	13 staff in DBT team. Provided all DBT components plus additional DBT related services. Team funding the equivalent of £520,000 per annum
Barley et al., 1993 [40]	America. Inpatient psychiatric hospital	Programme description	Naturalistic outcome evaluation	Reduced para-suicidal behaviour	Transitioned to a DBT model over a two year period

*Abbreviations:* *BPD* Borderline Personality Disorder, *CFIR* Consolidated Framework for advancing Implementation science, *DBT* Dialectical Behaviour Therapy, *DSH* Deliberate Self-harm, *QOL* Quality of Life, *TAU* Treatment As Usual, *UK* United Kingdom

### Evidence synthesis

Overall, 788 framework codes were assigned to the extracted data: 170 codes were allocated to studies specifically considering implementation, 209 codes to process analysis studies, 224 codes to programme descriptions, and the remainder were assigned to discussion papers (see Additional file 4). The DBT implementation framework is used to present the literature synthesis and, when possible, the data discussed is derived from the papers which explicitly studied implementation.

#### Context

Our initial DBT framework included seven context sub-elements (culture, leadership, evaluation, goal fit and suitability, individual characteristics, facilitative administrative supports and system interventions). Our synthesis of the literature yielded five primary sub-elements (culture, leadership, goal fit and suitability, facilitative administrative supports and system interventions), which are discussed below:

### Culture

There are two elements of culture that capture staff behaviour within the organisation [5, 6]:

#### Communication processes

Better ratings of organisation cohesion and communication correlated with the implementation of more DBT components [26], perhaps because institutional adoption of DBT depends on the collaboration of many staff [34]. On-going external consultation helps achieve sustainable programmes [23] and good communication was important within the DBT consultation team [29]. There were examples of communication forming both a facilitator (e.g. [40]) and a barrier [46]. Communication within and across teams seemed particularly important when client characteristics, such as intellectual disability or offender status, meant collaborative working was essential (e.g. [45]).

#### Climate

Higher scores on the Team Climate Inventory correlated with the implementation of more DBT components [26] and limited understanding of staff and patients’ needs could form a barrier [29]. The importance of team support was endorsed by therapists [28]. Attitudes toward BPD seemed key. A non-judgemental, validating stance seems necessary to create the right environment [25], and better attitudes towards BPD correlated with increased use of DBT [35]. In one survey negative administrator attitudes reportedly impeded implementation [23].

### Leadership

In a therapist survey, one of the most common reasons for DBT team cessation was a lack of leadership or organisation ‘buy-in’. Where team leadership was supportive, 19.6% of respondents said this facilitated implementation [31]. Respondents in another study similarly reported that a lack of understanding amongst service leaders constituted an implementation barrier [28]. Often the implementation of DBT had not been pre-planned and in these scenarios having a ‘DBT champion’ in the organisation seemed important. Champions needed to have influencing skills (e.g. [51]), cultural sensitivity, a willingness to undertake tasks, such as, securing funding [43], and an ability to model DBT skills [46]. In many cases the DBT consultation team seemed to undertake championing tasks through generating interest (e.g. [59]), establishing collaborations (e.g. [65]), offering expertise to other agencies (e.g. [52]) and providing support to the wider staff team (e.g. [69]).

### Goal fit and suitability

Sometimes DBT was viewed positively from the outset [36] and greater confidence in DBT effectiveness correlated with increased use of DBT [35]. However, DBT was not always seen as suitable [24, 30]. DBT implementation was also weakened by competing service priorities [31]. For instance, in a substance abuse service, DBT was incompatible with the delivery model of short visits primarily providing methadone [23]. Some administrators were concerned about the telephone coaching component of DBT, as telephone support had not worked previously [36] and services need a minimum number of patients to run DBT groups [30]. However, whilst belief in DBT suitability and fit could facilitate implementation (e.g. [58]), the lack of this belief was not necessarily a barrier, as perceptions could change during the implementation process (e.g. [55]).

### Facilitative administrative supports

Insufficient time could be a barrier, whereas the allocation of sufficient time could be a facilitator [31]: in one survey, 42% of therapists reported having a lack of time to provide DBT [23]. Some therapists talked about needing to divide their time between different tasks [28] and administrators were concerned that DBT training would keep staff from their clinical duties [36]. Other required resources were finances [36] and space: having adequate space correlated with the implementation of more DBT components [26]. The data also suggests that contingency management has the potential to influence implementation. For instance, organisations often failed to reduce other staff-held responsibilities to compensate for new DBT commitments [23] thereby punishing engagement in DBT. Enabling natural contingencies, such as, smaller caseloads and enabling staff to hold a highly visible role in the service seemed more effective (e.g. [40]) than providing tangible reinforcements (e.g. [65]) - although see [48] for an exception.

### System interventions

In the US services need to ensure they receive sufficient referrals to remain viable and so coordination with external agencies is necessary [36]. There were five examples of coalitions facilitating implementation ([14, 42, 54, 55, 59]). One research group suggested that training courses and merging consultation teams might foster coalitions [23] and there was an example of a service establishing two consultation teams: one service-led, the other interagency [45].

#### Evidence

Informed by PARIHS our initial framework referred to the sub-elements of research and published guidance, clinical experience and professional knowledge, preferences and experiences and local knowledge. However, our search yielded just two primary sub-elements (preferences and experiences and local knowledge and evaluation):

### Preferences and experiences

Some therapists expressed a preference for DBT [28] but 47% of therapists said there were challenges in recruiting sufficient patients [23]. Patients reported that they liked many aspects of DBT [29], though they need sufficient cognitive capacity to understand DBT skills and this may constitute a barrier for some [25]. The literature contained evidence that recruitment (e.g. [38]) and attrition (e.g. [52]) could be a problem and there were many attempts to reduce attrition including: ensuring participation was voluntary (e.g. [51]), careful selection of patients (e.g. [42]), providing more information about what DBT would entail (e.g. [53]) and, when appropriate, involving caregivers (e.g. [46]). On two occasions tangible reinforcement was offered [40, 64].

### Local knowledge and evaluation

Evidence of clinical improvement can reinforce implementation attempts [24], although only 7% of respondents in one survey agreed that improved patient outcomes were an implementation facilitator [31]. Sometimes demonstrating good patient outcomes generated interest in DBT [37] and led to further funding [43]. However, there were only five examples of services routinely evaluating outcomes ([37, 39, 42, 47, 48]).

#### Facilitation

Our initial framework referenced six sub-elements (strategies, support, training, coaching and ongoing consultation, facilitator skills/ qualities and recruitment and selection). However, our search and synthesis yielded two primary sub-elements (team capacity and commitment, and training and ongoing support):

### Team capacity and commitment

Some therapists thought the effectiveness of DBT was solely due to its techniques and theory [29], but this view was not universal. Several optimal therapist attributes were detailed including a stance of equality, an ability to synthesise validation and challenge, a good understanding of DBT skills, as well as, group management and teaching abilities [25]. Therapist confidence also seemed important [27] and this could be enhanced through DBT implementation [24, 28] and training [35]. Administrators selected staff based on their seniority and motivation and recruited to ensure team diversity [36]. Therapists’ academic qualifications seem less important [26], but they do need to be skilled clinicians [31].

Insufficient staffing can jeopardise sustainability ([23, 30, 36]) and staff turnover is a further barrier [23, 31]. For instance, in one prospective implementation study 55% of therapists remained working at their original organisation at two year follow-up [35]. A possible reason for retention problems is that new DBT therapists initially reported increased stress levels and there was a tendency (although this was not statistically significant) for staff burnout to occur over time [28]. A small association suggested that larger teams implement more DBT components [26]. Smaller teams are likely to operate within larger services, with staff having additional roles. These nested programmes seem common as several therapists reported working in the DBT team part-time [28] and contrary to the Ditty et al. [26] findings, there were examples of successful nested teams (e.g. [56]) and teams dependent on part-time staffing (e.g. [52]).

### Training and ongoing support

Clinicians from diverse disciplines can acquire a solid grounding in DBT through training [34]. Training facilitates implementation [31] and attending more training is associated with greater confidence and use of DBT [27]. For instance, training significantly increased the use of skills training, treatment targets, daily diary cards and dialectic strategies [35]. DBT knowledge also moderately correlated with all indices of training [34]. Unfortunately, limited feedback about training has been collected. Therapists reported that training enabled them to use DBT in their practice but they wanted more detailed instruction on how to perform specific interventions, such as conducting chain analysis of problem behaviour [23]. In one study, E-learning was most successful in increasing reported application of DBT [32]. In an earlier report, instructor-led training improved therapist self-efficacy and satisfaction but no method increased therapist skilfulness [33]. There was some evidence that training could improve clinical outcomes (e.g. [66]) but a lack of training was not always a barrier: graduate students with minimal training achieved 88% fidelity with DBT methods when facilitating skills groups [64].

On-going consultation is important [23, 36] and lacking access to a DBT consultation team can be an implementation barrier [27]. DBT consultation teams can help therapists achieve dialectical synthesis [25] and complement [25, 28] or compensate for lack of training [34]. Access to individual supervision is also important [26]: lack of supervision was the most frequently reported barrier to using DBT skills in one report [33] and in a UK survey [31], 34% of respondents said supervision facilitated the use of DBT. Limited feedback has been collected about supervision experiences: therapists reported that supervision increases both stress and coping [28].

#### DBT

Our initial framework identified four sub-elements related to the intervention (design quality and packaging, adaptability, complexity and costs). Our search and synthesis yielded sub-elements related to the design quality, packaging and costs:

### Design quality, packaging and costs

DBT can be a complex therapy to implement: several DBT skills can be difficult to understand and apply [25] and trainers have reported that therapists have difficulty applying DBT’s behavioural components [34]. Aspects of DBT which seem important are its treatment contract emphasising shared responsibility [29] and its adaptability [24, 30]. For instance, despite some authors believing that DBT’s manual-based nature is important [29, 34], there were many examples of adaptations (e.g. [48]) with adjustments often altering how telephone skills coaching was provided (e.g. [67]). In the US, limited reimbursement is a barrier to implementing DBT [27, 36] and in the UK, 29% of survey respondents said that allocating sufficient finances to DBT delivery was an implementation facilitator [31].

#### Implementation process

CFIR separates the implementation process into sub-elements related to execution, engagement, planning, evaluation and reflection. It was not possible to dissect these individual components in the literature. However, there were two examples of clearly executed implementation plans [40, 46] and five examples of services forming teams to oversee the implementation process ([41, 49, 51, 57, 59]). A lack of an implementation plan can be an implementation barrier [31] but plans do not guarantee success. For instance, one study planned to introduce a number of resources (e.g., demonstration videos, an online forum and telephone consultation) to improve DBT adherence during implementation [23]. During the study there were no requests for consultation and in post-implementation interviews therapists did not refer to any of the available resources. This study highlights that providing resources alone is unlikely to promote implementation.

## Discussion

**Fig. 3:**
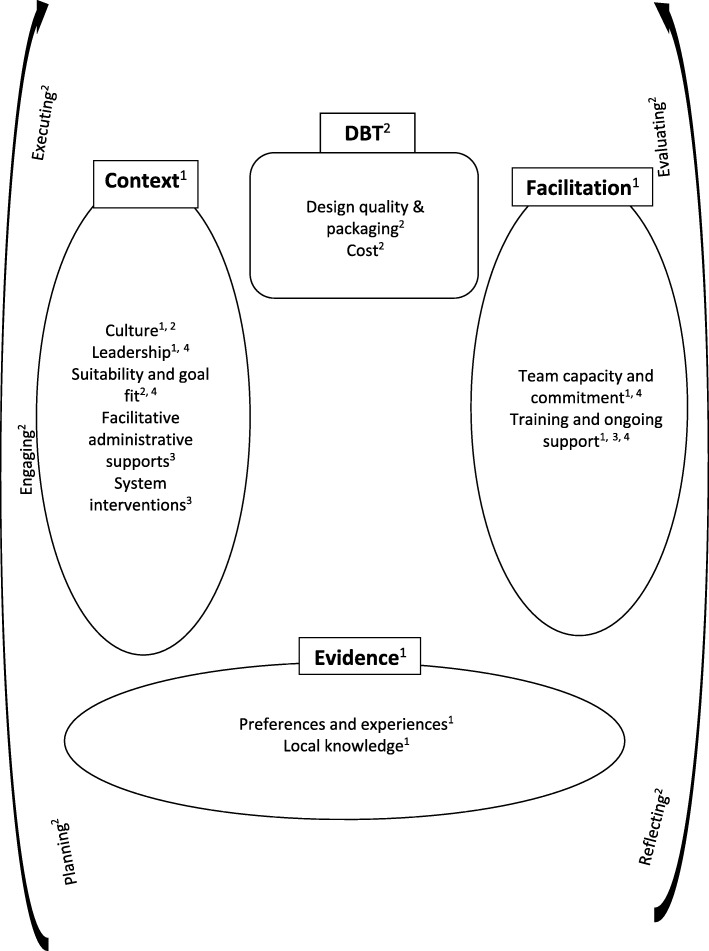
Revised DBT implementation framework. Key: PARIHS, 2004^1^; Damschroder et al., 2009^2^; Fixsen & Blasé, 2009^3^, Swales 2010a, 2010b^4^

This critical literature review synthesised the DBT implementation literature to refine a DBT implementation framework. The framework sufficiently captured the data and no new elements or sub-elements were required (see Additional file 4). However, some refinements were made to create a more parsimonious and relevant framework for DBT. For instance, coding indicated that some sub-elements were capturing similar data. For example, the sub-elements ‘individual characteristics’, ‘facilitator skills/ qualities’, and ‘recruitment and selection’ were re-conceived into a sub-element called team capacity and commitment. Additionally, some sub-elements arose infrequently in the literature (e.g., research and published guidance) and these were therefore omitted (the refined framework is illustrated in Fig. 3). However, we acknowledge that limited literature on an implementation barrier is not necessarily evidence that the barrier is not significant in DBT. For instance, cost may prevent both implementation and research meaning that the magnitude of barriers related to cost may not be sufficiently reflected in the framework, as the literature primarily reflects successfully funded work.

The utility of transdisciplinary implementation frameworks, such as PARIHS [5, 6], is highlighted by these findings: elements primarily derived from existing frameworks effectively captured DBT implementation barriers and facilitators. The critical review process also proved to be useful in guiding the framework refinement and the synthesis of the literature. However, only 14 papers were retrieved that specifically focused on DBT implementation and this suggests that a DBT implementation framework may usefully underscore the most important considerations for DBT implementers.

The DBT implementation framework is a useful resource for DBT practitioners and service leaders who are planning (or overseeing current) DBT implementations. The synthesis indicates that implementers should consider the following recommendations:

- When introducing DBT into practice clinicians and organisations should encourage the staff team to operate a benign approach to BPD and ensure there are good communication systems in place.
- When establishing a DBT team, it seems important to recruit therapists with sufficient cognitive flexibility, whose personal qualities align with those espoused by DBT, such as, having a non-judgemental stance.
- The DBT team will benefit from on-going supervision and consultation and therapists should receive adequate training.
- Leadership support is beneficial and in situations where implementation is not pre-planned, a DBT champion can help.
- It is beneficial for services to evaluate whether DBT needs adapting to suit their organisation.

Despite the apparent strengths of the DBT implementation framework, the limitations of the literature need to be taken into account. The framework’s generalisability cannot be ascertained as the reviewed literature only provided information about implementation in Western contexts and primarily reported on implementation in statutory outpatient mental health services. The most commonly retrieved papers were trial reports and implementing DBT in a research context may have significant differences from implementation in clinical services. Furthermore, the decision to include only published literature biased the review towards considering effective DBT implementations as most trials and programme descriptions reported positive results. In particular the literature reviewed, with the exception of a DBT implementation with Native Americans [43], cannot inform how DBT implementation is achieved with marginalised and particularly high risk populations, such as cultural minority groups. When more information about DBT implementations with such populations become available, the framework may require refinement.

The implementation papers reviewed also had methodological limitations. Most data was collected retrospectively and relied on self-reports of implementation success (e.g. [26]). Samples may not have been representative, for instance, the response rate in one study was approximately 14% [30]. Furthermore, survey instruments had not always been validated (e.g. [35]) and most quantitative data was correlational (e.g. [26]), so causation could not be inferred.

Limitations in the literature and framework provide opportunities for future research. It is acknowledged that interrater agreement when using the framework to code data could be improved. The current framework is sufficiently detailed for use by DBT practitioners and service leaders who are planning implementation, but in a research and academic context one next step will be to develop more precise definitions of some sub-elements. Although, the current literature cannot inform how implementation barriers and facilitators interact or how they are weighted in different contexts, a few tentative potential relationships warrant further exploration. For example, communication and contingency management might be particularly important in organisations providing team approaches, such as, inpatient services. Access to on-going support may be particularly important if staff have not received comprehensive DBT training. The complexity of DBT may only form a barrier if clients and staff have not been appropriately selected. To refine and further develop the DBT implementation framework in the academic context, another next step will be to undertake further research to explore these tentative ideas about how the framework elements interact and are weighted. To explore these relationships further, prospectively collected data will be needed as is planned in the EMPOWER research programme (NIHR Programme Grant: RP-PG-1212-20,011).

## Conclusions

This review has explored the DBT implementation literature and developed a bespoke framework to inform future implementations. The literature synthesis has highlighted some important implementation considerations but prospective DBT implementation studies are now needed to explore the relative weighting of and relationships amongst these barriers and facilitators.

## Additional files

Additional file 1: A detailed description of the DBT implementation framework. Describes the DBT implementation framework and its elements in greater detail. (DOCX 22 kb)

Additional file 2: Critical literature review: example search. Provides the literature search used in the CINAHL (EBSCO) database. (DOCX 12 kb)

Additional file 3: Data extraction template. The data extraction form used in the critical literature review. (DOCX 12 kb)

Additional file 4: DBT implementation framework: overview of assigned codes. Illustrates how framework elements and sub-elements were operationalised into codes. Lists which codes were assigned by paper and study type. Also provides a tally of how many times each code was assigned. (DOCX 30 kb)

## Abbreviations

BPD: Borderline Personality Disorder
CFIR: Consolidated Framework for Implementation Research
DBT: Dialectical Behaviour Therapy
DBT-SE: Dialectical Behaviour Therapy- Skills for Employment
EMPOWER: Enabling and Motivating people (with a Personality Disorder) in Occupation, Wellbeing, Education and Responsibility
PARIHS: Promoting Action on Research Implementation in Health Services
