# Supplementary material for: The development and theoretical application of an implementation framework for dialectical behaviour therapy: a critical literature review
Source: Borderline Personal Disord Emot Dysregul. 2019 Feb 12;6:2. doi: 10.1186/s40479-019-0102-7 (PMC6373034; doi:10.1186/s40479-019-0102-7)
Supplement: Supplementary file 1 — A detailed description of the DBT implementation framework. Describes the DBT implementation framework and its elements in greater detail. (DOCX 22 kb) [file 40479_2019_102_MOESM1_ESM.docx]

**A detailed description of the DBT implementation framework**

*Context*

Context can be defined as “the set of circumstances or unique factors that surround a particular implementation effort” [1]. In Promoting Action on Research Implementation in Health Services (PARIHS), context relates to the environment or intervention setting and the focus is on three main sub-elements:

*Culture*

How organisations (the intervention setting) contribute to successful implementation is often overlooked [2]. Understanding the culture (the way things are done) within organisations can help to identify implementation barriers and enablers. In the augmented PARIHS model (called the Consolidated Framework for Implementation Research (CFIR) model), culture is highlighted within the “inner” setting domain to describe the norms, values, and assumptions of an organization [1]. Critical factors include those which are changeable, provide a buffer for resistance, or enhance desired behaviours [3].

*Leadership*

Leaders can influence organisation culture, and within PARIHS the relationship between leadership and culture is made explicit [4]. In the revised PARIHS model the focus is on leadership support for implementation, rather than leadership per se [3]. This support can be provided through the use of management behaviour that clarifies staff roles, shows visible support for implementation, supports change within the organisation and ensures effective communication [3]. Leadership style and how DBT-SE is championed may also influence the success of implementation [5].

*Evaluation*

When PARIHS was revised, the sub-element *measurement* was changed to *evaluation* to reflect the fact that multiple approaches and sources of feedback should be incorporated into an organisation’s evaluative mechanisms [6]. It is suggested that evaluation should extend across individual, team and systems levels and use a range of different methods (for example; clinical audit and performance appraisal [7]) to facilitate the timely use of evaluation data in practice [8]. From the augmented PARIHS model further sub-elements were incorporated:

*Suitability and goal fit*

‘Suitability and goal fit’ from CFIR highlight that the provider’s perception of the intervention is important [1]. The Core Implementation Components model referred to this as ‘organisation characteristics’ [9] but the term ‘suitability’ was selected (derived from the DBT literature) as it is more specific to implementing an intervention. Within this sub-element it may be essential to pay attention to the needs and goals of the organisation where implementation is planned [9], including the organisational readiness to change. Some DBT implementers have argued that organisations should be assisted to weigh up the pros and cons of implementation and arrive at a commitment to implement or not [5]. The outcome of this deliberation process will influence the organisational readiness for change [10], i.e. “the extent to which organizational members are psychologically and behaviourally prepared to implement organizational change” [11]. Readiness for change can be assessed at the individual, team or organisational level [10].

*Individual characteristics*

Examining ‘individual characteristics’ is important, as in the implementation process, no-one is a passive recipient [12] [13]: “setting and intervention constructs are rooted, ultimately, in the actions and behaviours of individuals” [1]. Examining individual characteristics can shed light on how people influence the implementation process.

*Additional sub-elements*

The incorporation of ‘facilitative administrative supports’, a term derived from the Core Implementation Components model [14] acknowledges that it is often the interaction between organisation behaviour and practitioner perception of this, which can reinforce or mitigate against implementation. For example, if a service expects practitioners to undertake additional data collection and conduct new interventions on top of their existing caseloads, then it is likely that practitioners will feel burdened by implementation initiatives. Finally, the idea of ‘system interventions’ from the Core Implementation Components model [14] refers to the strategies that the organisation uses when working with external systems (such as study teams and other service providers).

*Evidence*

In the PARIHS framework, evidence is interpreted as including both propositional and non-propositional forms of knowledge [8], reflecting the idea that knowledge can be just learnt or learnt *and* used. Within PARIHS, this is represented by four sub-elements:

*Research and published guidance*

Evidence is often most persuasive if it is published research, devoid of controversy [3]. Receptiveness to published research may vary according to stakeholder group. For example, clinicians may view such evidence as undermining their autonomy or as irrelevant [15]. In contrast, service leaders and commissioners might favour published evidence as the basis for decision making, as they have to comply with policies mandating evidence-based practice.

*Clinical experience and professional knowledge*

Clinical experience and professional knowledge may influence many aspects of implementation, such as, motivation, leadership support and how DBT is championed. Previous research suggests that clinicians may lack confidence in supporting people with Borderline Personality Disorder (a common client group for DBT interventions) and desire further training [16].

*Preferences and experience*

This sub-element has some overlap with ‘individual characteristics’ (a sub-element of context). However, ‘individual characteristics’ is a broader construct referring to the beliefs, attitude and behaviour of all stakeholders. Preferences and experiences, in comparison, has a focus on the views of intervention recipients.

*Local knowledge*

Within PARIHS, data and information collected by the organisation is included only if it is found to be valued, has been collected and analysed in a systematic way, and is evaluated and reflected upon [8]. This is different in focus from the ‘evaluation’ sub-element of context, which refers to all organisational data collection and feedback mechanisms.

*Facilitation*

Facilitation is a multifaceted term and the definition “enabling (making easier) the implementation of evidence into practice” [17] guided the selection of sub-elements.

*Strategies*

Strategies to implement new interventions are conceived to be multifaceted in PARIHS [17]. The DBT literature indicates some particular strategies that may be employed, such as an organisational pre-treatment approach [2]. Strategies are often explored to see if they “are replicable, how these attributes are created and the characteristics of environments in which they are worth replicating” [18].

*Support*

This refers to how the organisation assists facilitators, such as providing networking and advice. PARIHS highlights training as a specific form of support and we have included this as a separate sub-element.

*Facilitator skills and qualities*

Facilitators are integral to the success or failure of the implementation process, as they can affect the context of implementation and the application of evidence [8]. Facilitation “involves the facilitator working with individuals, teams, and organisations to prepare, guide, and support them through the implementation process” [19]. PARIHS suggests attending to the skills and qualities that facilitators bring to implementation efforts. Within PARIHS, the ability of the implementation facilitator to be flexible and adjustable is clearly associated with skill level [8] [3].

*Training, Coaching and ongoing consultation*

PARIHS suggests considering ongoing education and the Core Implementation Components model considers the professional development opportunities provided by the organisation [14]. To make this sub-element more DBT specific, we used the label ‘coaching and ongoing consultation’. The DBT literature indicates that the availability and quality of supervision (which can enhance facilitator confidence, emotional regulation and clinical decision making) may be important.

*Additional sub-elements*

When DBT is team-based, the mix of skills in the team is important [5] and this involves a consideration of the ‘recruitment and selection’ methods used to create the team.

*DBT*

In the augmented PARIHS model [1], notable intervention elements are characteristics of the intervention, other than its content and purpose, which have the potential to affect the success of implementation.

*Design quality and packaging*

CFIR considers several intervention features [1], such as, the intervention ‘design quality and packaging’, which includes the history and origins of the intervention [1]. It also incorporates how the intervention is presented to the organisation.

*Adaptability*

The term ‘adaptability’ was selected to refer to a series of related ideas regarding intervention trial ability and interventions having adaptable peripheries [1]. There is already some discussion in the DBT literature about the relative merits of DBT being a manualised therapy, whilst being adaptable to different circumstances.

*Complexity and Cost*

Informed by CFIR the framework included sub-elements relating to ‘complexity’ and ‘cost’. Complex interventions may be more complicated to implement [1] and expensive interventions may be prohibitive for some organisations.

*Implementation process*

CFIR, the augmented PARIHS model, indicates that the process of planning, engaging, executing, and reflecting/evaluating is essential to implementation [1]. This can encompass how organisational needs and preferences are taken into account and the degree of flexibility and tailoring made to meet individual needs within the organisation [1]. It also includes a consideration of implementation fidelity, such as, if there have been unintended deviations from the implementation plan.

**References**

[1] Damschroder LJ, Aron DC, Keith RE, Kirsh SR, Alexander JA, Lowery JC. Fostering implementation of health services research findings into practice: a consolidated framework for advancing implementation science. Implement Sci. 2009; 4:50. Doi: 10.1186/1748-5908-4-50

[2] Swales MA. Implementing Dialectical Behaviour Therapy: organizational pre-treatment. Cogn Behav Therapist. 2010b; 3: 145–157. Doi: 10.1017/S1754470X10000115

[3] Stetler CB, Damschroder LJ, Helfrich CD, Hagedorn HJ. A guide for applying a revised version of the PARIHS framework for implementation. Implement Sci. 2011; 6:99. Doi: 10.1186/1748-5908-6-99

[4] McCormack B, Kitson A, Harvey G, Rycroft-Malone J, Titchen A, Seers K. Getting evidence into practice: the meaning of ‘context’. J Adv Nurs. 2002; 38: 94–104. Doi: 10.1046/j.1365-2648.2002.02150.x

[5] Swales MA. Implementing DBT: selecting, training and supervising a team. Cogn Behav Therapist. 2010a; 3: 71-79. Doi: 10.1017/S1754470X10000061

[6] Rycroft-Malone J, Kitson A, Harvey G, McCormack B, Seers K, Titchen A, et al. Ingredients for change: revisiting a conceptual framework. Qual Saf Health Care. 2002; 11: 174–180. Doi: 10.1136/qhc.11.2.174

[7] Rycroft-Malone J. The PARIHS framework—a framework for guiding the implementation of evidence-based practice. J Nurs Care Qual. 2004; 19: 297-304. Doi: 10.1097/00001786-200410000-00002

[8] Rycroft-Malone J. Promoting action on research implementation in health services (PARIHS). In Rycroft-Malone J, Bucknall, T (Eds). Models and frameworks for implementing evidence-based practice: linking evidence to action (pp.109-136). Singapore: Wiley-Blackwell, Sigma Theta Tau International; 2010

[9] Fixsen DL, Naoom SF, Blase KA, Friedman RM, Wallace F. Implementation research: a synthesis of the literature. Tampa FL: University of South Florida, Louis de la Parte Florida Mental Health Institute, The National Implementation Research Network (FMHI Publication #231); 2005

[10] Shea CM, Jacobs SR, Esserman DA, Bruce K, Weiner BJ. Organizational readiness for implementing change: a psychometric assessment of a new measure. Implement Sci. 2014; 9:7. Doi: 10.1186/1748-5908-9-7

[11] Weiner BJ, Amick H, Lee SY. Conceptualization and measurement of organizational readiness for change: a review of the literature in health services research and other fields. Med Care Res Rev. 2008; 65: 379-436. Doi: 10.1177/1077558708317802

[12] Greenhalgh T, Robert G, Bate P, Kyriakidou O, Macfarlane F, Peacock R. How to spread good ideas –a systematic review of the literature on diffusion, dissemination and sustainability of innovations in health service delivery and organisation. London, UK: National Co-ordinating Centre for NHS Service Delivery and Organisation R&D (NCCSDO); 2004

[13] Rycroft-Malone J, Seers K, Chandler J, Hawkes CA, Cricthon N, Allen C, et al. The role of evidence, context, and facilitation in an implementation trial: implications for the development of the PARIHS framework. Implement Sci. 2013; 8:28. Doi: 10.1186/1748-5908-8-28

[14] Fixsen DL, Blase KA, Naoom SF, Wallace F. Core implementation components. Res Social Work Prac. 2009; 19: 531-540. Doi: 10.1177/1049731509335549

[15] National Institute for Health and Clinical Excellence. How to change practice. Understanding, identifying and overcoming barriers to change. London, UK: NICE; 2007

[16] Ogrodniczuk JS, Kealy D, Howell-Jones G. A view from the trenches: a survey of Canadian clinicians' perspectives regarding the treatment of borderline personality disorder. J Psychiatr Pract. 2009; 15: 449-453. Doi: 10.1097/01.pra.0000364286.63210.db

[17] Harvey G, Loftus-Hills A, Rycroft-Malone J, Titchen A, Kitson A, McCormack B, et al. Getting evidence into practice: the role and function of facilitation. J Adv Nurs. 2002; 37: 577–588. Doi: 10.1046/j.1365-2648.2002.02126.x

[18] Winter SG, Szulanski G. Replication as strategy. Organ Sci. 2001; 12: 730-743. Doi: 10.1287/orsc.12.6.730.10084

[19] Seers K, Cox K, Crichton NJ, Edwards RT, Eldh AC, Eastabrooks CA, et al. FIRE (facilitating implementation of research evidence): a study protocol. Implement Sci. 2012; 7:25. DOI: 10.1186/1748-5908-7-25
