# Supplementary material for: The development and theoretical application of an implementation framework for dialectical behaviour therapy: a critical literature review
Source: Borderline Personal Disord Emot Dysregul. 2019 Feb 12;6:2. doi: 10.1186/s40479-019-0102-7 (PMC6373034; doi:10.1186/s40479-019-0102-7)
Supplement: Supplementary file 2 — Critical literature review: example search. Provides the literature search used in the CINAHL (EBSCO) database. (DOCX 12 kb) [file 40479_2019_102_MOESM2_ESM.docx]

**Critical literature review: example search**

CINAHL (EBSCO) database:

1. CINAHL Keyword search: Dialectical Behaviour Therapy
2. Title/ Abstract search: “Dialectical Behaviour Therap*” OR “Dialectical Behavior Therap*”
3. #1 OR #2
4. CINAHL Heading (unable to explode): Implementation
5. Title/ Abstract search: Implement* OR “Process analys*”
6. #4 OR #5
7. #3 AND #6
8. English limiter applied
9. Peer review published limiter applied
