# Supplementary material for: The development and theoretical application of an implementation framework for dialectical behaviour therapy: a critical literature review
Source: Borderline Personal Disord Emot Dysregul. 2019 Feb 12;6:2. doi: 10.1186/s40479-019-0102-7 (PMC6373034; doi:10.1186/s40479-019-0102-7)
Supplement: Supplementary file 3 — Data extraction template. The data extraction form used in the critical literature review. (DOCX 12 kb) [file 40479_2019_102_MOESM3_ESM.docx]

**Data extraction template**

| **Reference** |  |
| --- | --- |
| **Design** |  |
| **Context** |  |
| **Methodology** |  |
| **Implementation questions asked** *if survey or interview* |  |
| **Study limitations** |  |
| **Findings** |  |
| **Intervention** *if not standard DBT* |  |
| **Implementation barriers identified** |  |
| **Implementation facilitators identified** |  |
| **Conclusions/ author recommendations** |  |
