# Supplementary material for: The development and theoretical application of an implementation framework for dialectical behaviour therapy: a critical literature review
Source: Borderline Personal Disord Emot Dysregul. 2019 Feb 12;6:2. doi: 10.1186/s40479-019-0102-7 (PMC6373034; doi:10.1186/s40479-019-0102-7)
Supplement: Supplementary file 4 — DBT implementation framework: overview of assigned codes. Illustrates how framework elements and sub-elements were operationalised into codes. Lists which codes were assigned by paper and study type. Also provides a tally of how many times each code was assigned. (DOCX 30 kb) [file 40479_2019_102_MOESM4_ESM.docx]

**DBT implementation framework: overview of assigned codes**

| **Framework sub-elements** | **Individual codes contributing to sub-element** | **Total number of codes assigned** | **Papers from which code total derived** | | | |
| --- | --- | --- | --- | --- | --- | --- |
|  |  |  | **Implementation** | **Process analysis** | **Programme description** | **Discussion** |
| ***Context*** | | | | | | |
| Culture | | | | | | |
|  | Organisation characteristics | 12 | Ditty et al., 2015 [26]  Perseius et al., 2003 [29]  Swales et al., 2012 [31]  Herschell et al., 2014 [35] | Blennerhassett et al., 2009 [54] | Barley et al., 1993 [40]  Sampl et al., 2010 *x 2* [48] | Swales, 2010b [12]  Berzins & Trestman, 2004 [14]  Chugani, 2015 [16]  Swenson et al., 2002 [21] |
|  | Communication | 24 | Carmel et al., 2014 [23]  Ditty et al., 2015 *x 2* [26]  Perseuis et al., 2003 [29]  Hawkins & Sinha, 1998 [34] | Blennerhassett et al., 2009 [54]  Comtois et al., 2007 [57]  Eccleston & Sobello, 2002 *x 2* [58]  Hjalmarsson et al., 2008 *x 2* [59]  Nee & Farman, 2005 [63]  Koons et al., 2006 [65]  Prendergast & McCausland, 2007 [67]  Woodberry & Popenoe, 2008 [71] | APA Gold Award, 2004 [37]  Baillie & Slater, 2014 [39]  Barley et al., 1993 *x 2* [40]  Lew et al., 2006 [45]  Little et al., 2010 [46]  Sampl et al., 2010 [48]  Wolpow et al., 2000 [51] | McHugh & Barlow, 2010 [19] |
|  | Climate (including identification with the organisation) | 23 | Cunningham et al., 2004 [25]  Ditty et al., 2015 [26]  Perseuis et al., 2007 [28]  Perseius et al., 2003 [29]  Herschell et al., 2014 [35] | Hjalmarsson et al., 2008 [59] | Engle et al., 2013 *x 2* [42]  Little et al., 2010 *x 2* [46]  Morrissey & Ingamells, 2011 [47]  Sunseri, 2004 [49] | Swales, 2010a *x 3* [11]  Swales 2010b [12]  Huffman et al., 2003 *x 3* [17]  Koerner, 2013 *x 4* [18] |
|  | Approach to BPD | 16 | Carmel et al., 2014 *x 2* [23]  Cunningham et al., 2004 [25]  Perseius et al., 2003 *x 2* [29]  Herschell et al., 2014 *x 2* [35] | Hjalmarsson et al., 2008 [59] | Baillie & Slater, 2014 [39] | Huffman et al., 2003 *x 2* [17]  Koerner, 2013 *x 2* [18]  Swenson et al., 2002 *x 3* [21] |
| Leadership | | | | | | |
|  | Leadership | 26 | Perseuis et al., 2007 [28]  Swales et al., 2012 *x 4* [31]  Herschell et al., 2014 [35] | Ben-Porath et al., 2004 [53]  Comtois et al., 2007 *x 2* [57]  Hjalmarsson et al., 2008 [59]  Koons et al., 2006 [65]  Van den Bosch et al., 2002 [70] | APA Gold Award, 2004 [37]  Arroyo et al., 2012 [38]  Barley et al., 1993 *x 2* [40]  Gold Award, 1998 *x 2* [41]  Engle et al., 2013 [42]  Lew et al. 2006 [45]  Little et al., 2010 [46]  Sampl et al., 2010 [48]  Wolpow et al., 2000 [51] | Swales, 2010a [12]  Swenson et al., 2002 *x 2* [21] |
|  | Champions | 34 |  | Ben-Porath et al., 2004 [53]  Blennerhassett et al., 2009 [54]  Comtois et al., 2007 [57]  Hjalmarsson et al., 2008 [59]  Katz et al., 2004 [61]  Koons et al., 2006 [65]  Prendergast & McCausland, 2007 [67]  Trupin et al., 2002 [69]  Woodberry & Popenoe, 2008 [71] | APA Gold Award, 2004 *x 2* [37]  Baillie & Slater, 2014 [39]  Gold Award, 1998 [41]  Engle et al., 2013 *x 2* [42]  Kinsey & Reed, 2015 *x 3* [43]  Lew et al., 2006 [45]  Little et al., 2010 [46]  Sampl et al., 2010 [48]  Sunseri, 2004 *x 2* [49]  Wolpow et al., 2000 [51]  Zinkler et al., 2007 [52] | Swales, 2010a *x 2* [11]  Swales, 2010b *x 2* [12]  Huffman et al., 2003 [17]  Koerner, 2013 [18]  McHugh & Barlow, 2010 [19]  Swenson et al., 2002 *x 2* [21] |
| Suitability | | | | | | |
|  | Suitability | 18 | Chwalek & McKinney, 2015 *x 2* [24]  Fredrick & Comtois, 2006 [27]  Perseius et al., 2003 [29]  Sharma et al., 2007 [30]  Herschell et al., 2014 *x 2* [35]  Herschell et al., 2009 *x 2* [36] | Eccleston & Sobello, 2002 [58]  Hjalmarsson et al., 2008 [59]  Van den Bosch et al., 2002 [70] | Barley et al., 1993 [40]  Kinsey & Reed, 2015 [43]  Little et al., 2010 [46]  Wolpow et al., 2000 [51] | Burroughs & Somerville, 2013 *x 2* [15] |
|  | Goal fit | 45 | Carmel et al., 2014 [23]  Sharma et al., 2007 [30]  Swales et al., 2012 [31]  Herschell et al., 2009 *x 2* [36] | Bohus et al., 2000 [55]  Comtois et al., 2007 [57]  Eccleston & Sobello, 2002 [58]  Hjalmarsson et al., 2008 [59]  James et al., 2015 *x 3*  [60]  Kerr et al., 2009 [62]  Nelson-Gray et al., 2006 [64]  Prendergast & McCausland, 2007 [67]  Rathus & Miller, 2002 [68]  Van den Bosch et al., 2002 [70]  Woodberry & Popenoe, 2008 [71] | Arroyo et al., 2012 [38]  Barley et al., 1993 *x 2* [40]  Gold Award, 1998 [41]  Engle et al., 2013 [42]  Lew et al., 2006 [45]  Little et al., 2010 *x 2* [46]  Morrissey & Ingamells, 2011 *x 2* [47]  Sunseri, 2004 [49]  Vitacco & Van Rybroek, 2006 [50]  Wolpow et al., 2000 *x 2* [51]  Zinkler et al., 2007 [52] | Swales, 2010b *x 4* [12]  Berzins & Trestman, 2004 *x 3* [14]  Burroughs & Somerville, 2013 *x 2* [15]  McHugh & Barlow, 2010 [19]  Scheel, 2000 [20]  Swenson et al., 2002 [21] |
| Facilitative administrative supports | | | | | | |
|  | Contingency management | 30 | Carmel et al., 2014 *x 2* [23]  Fredrick & Comtois, 2006 [27]  Sharma et al., 2007 [30]  Dimeff et al., 2011 [32] | Ben-Porath et al., 2004 [53]  Blennerhassett et al., 2009 [54]  Brassington & Krawitz, 2006 [56]  Hjalmarsson et al., 2008 *x 2* [59]  Rathus & Miller, 2002 [68]  Trupin et al., 2002 *x 4* [69]  Van den Bosch et al., 2002 [70] | Barley et al., 1993 [40]  Engle et al., 2013 *x 2* [42]  Lajoie et al., 2011 [44]  Little et al., 2010 [46]  Morrissey & Ingamells, 2011 [47]  Sampl et al., 2010 [48] | Swales, 2010b [12]  Huffman et al., 2003 [17]  Koerner, 2013 [18]  Swenson et al., 2002 *x 3* [21]  Swenson, 2000 [22] |
|  | Resources | 35 | Carmel et al., 2014 *x 2* [23]  Ditty et al., 2015 [26]  Fredrick & Comtois, 2006 [27]  Perseuis et al., 2007 [28]  Swales et al., 2012 *x 4* [31]  Herschell et al., 2009 [36] | Blennerhassett et al., 2009 *x 2* [54]  Brassington & Krawitz, 2006 [56]  Eccleston & Sobello, 2002 *x 2* [58]  Hjalmarsson et al., 2008 [59]  Nee & Farman, 2005 [63]  Pasieczny & Connor, 2011 [66]  Prendergast & McCausland, 2007 [67] | Arroyo et al., 2012 [38]  Engle et al., 2013 *x 2* [42]  Lajoie et al., 2011 [44]  Little et al., 2010 [46]  Morrissey & Ingamells, 2011 [47]  Zinkler et al., 2007 [52] | Swales, 2010a [11]  Berzins & Trestman, 2004 [14]  Burroughs & Somerville, 2013 *x 3* [15]  Huffman et al., 2003 [17]  Scheel, 2000 [20]  Swenson et al., 2002 *x 2* [21] |
| System interventions | | | | | | |
|  | Coalitions | 23 | Carmel et al., 2014 *x 3* [23]  Herschell et al., 2009 [36] | Blennerhassett et al., 2009 [54]  Bohus et al., 2000 [55]  Hjalmarsson et al., 2008 [59]  Nee & Farman, 2005 [63]  Nelson-Gray et al., 2006 [64]  Rathus & Miller, 2002 [68] | APA Gold Award, 2004 [37]  Engle et al., 2013 [42]  Kinsey & Reed, 2015 *x 2* [43]  Lew et al., 2006 *x 2* [45]  Morrissey & Ingamells, 2011 *x 2* [47] | Berzins & Trestman, 2004 [14]  Scheel, 2000 [20]  Swenson et al., 2002 *x 2* [21]  Swenson, 2000 [22] |
| ***Evidence*** | | | | | | |
| Research and published guidance | | | | | | |
|  | Research evidence | 8 |  | Bohus et al., 2000 [55]  Prendergast & McCausland, 2007 [67] | Morrissey & Ingamells, 2011 [47] | Swales, 2010b [12]  Berzins & Trestman, 2004 [14]  Swenson et al., 2002 *x 2* [21]  Swenson, 2000 [22] |
| Clinical experience and professional knowledge | | | | | | |
|  | Professional knowledge | 10 | Sharma et al., 2007 *x 2* [30]  Dimeff et al., 2011 [32]  Dimeff et al., 2009 [33]  Hawkins & Sinha, 1998 *x 3* [34]  Herschell et al., 2014 [35] | Bohus et al., 2000 [55] |  | Koerner, 2013 [18] |
| Preferences and experiences | | | | | | |
|  | Preferences | 28 | Chwalek & McKinney, 2015 *x 4* [24]  Cunningham et al., 2004 [25]  Fredrick & Comtois, 2006 [27]  Perseuis et al., 2003 *x 3* [29] | Bohus et al., 2000 [55]  Nelson-Gray et al., 2006 [64]  Van den Bosch et al., 2002 [70] | Baillie & Slater, 2014 *x 3* [39]  Barley et al., 1993 *x 2* [40]  Gold Award, 1998 [41]  Kinsey & Reed, 2015 [43]  Little et al., 2010 *x 2* [46]  Sunseri, 2004 *x 2* [49] | Scheel, 2000 [20]  Swenson et al., 2002 *x 4* [21] |
|  | Attrition/ retention | 41 | Carmel et al., 2014 [23]  Sharma et al., 2007 [30] | Ben-Porath et al., 2004 *x 2* [53]  Blennerhassett et al., 2009 *x 2* [54]  Eccleston & Sobello, 2002 *x 2* [58]  Hjalmarsson et al., 2008 [59]  James et al., 2015 [60]  Nee & Farman, 2005 [63]  Nelson-Gray et al., 2006 *x 6* [64]  Koons et al., 2006 [65]  Prendergast & McCausland, 2007 [67]  Van den Bosch, 2002 [70] | Arroyo et al., 2012 [38]  Baillie & Slater, 2014 *x 4* [39]  Barley et al., 1993 [40]  Gold Award, 1998 [41]  Engle et al., 2013 *x 4* [42]  Little et al., 2010 *x 3* [46]  Morrissey & Ingamells, 2011 [47]  Sunseri, 2004 [49]  Vitacco & Van Rybroek, 2006 [50]  Wolpow et al., 2000 *x 2* [51]  Zinkler et al., 2007 [52] | Swenson et al., 2002 [21] |
| Local Knowledge^$^ | | | | | | |
|  | Local knowledge | 11 | Chwalek & McKinney, 2015 [24]  Swales et al., 2012 [31] | Brassington & Krawitz, 2006 [56] | APA Gold Award, 2004 [37]  Baillie & Slater, 2014 [39]  Gold Award, 1998 [41]  Kinsey & Reed, 2015 [43]  Little et al., 2010 [46]  Zinkler et al., 2007 [52] | Swales, 2010b [12]  Berzins & Trestman, 2004 [14] |
|  | Evaluation | 15 |  | Rathus & Miller, 2002 [68] | APA Gold Award, 2004 [37]  Baillie & Slater, 2014 [39]  Engle et al., 2013 *x 2* [42]  Little et al., 2010 *x 2* [46]  Morrissey & Ingamells, 2011 [47]  Sampl et al., 2010 [48] | Swales, 2010b [12]  Chugani, 2015 *x 3* [16]  McHugh & Barlow, 2010 [19]  Swenson et al., 2002 [21] |
| ***Facilitation*** | | | | | | |
| Team capacity and commitment | | | | | | |
|  | Facilitator qualities | 13 | Fredrick & Comtois, 2006 [27]  Perseuis et al., 2007 [28]  Perseuis et al., 2003 *x 2* [29]  Swales et al., 2012 [31]  Herschell et al., 2014 [35] | Hjalmarsson et al., 2008 [59]  Pasieczny & Connor, 2011 [66]  Van den Bosch et al., 2002 [70] | Arroyo et al., 2012 [38]  Little et al., 2010 [46]  Sunseri, 2004 [49] | Berzins & Trestman, 2004 [14] |
|  | Individual characteristics | 13 | Perseuis et al., 2007 [28]  Hawkins & Sinha, 1998 [34] | Trupin et al., 2002 [69] | Little et al., 2010 *x 2* [46]  Sunseri, 2004 *x 2* [49]  Wolpow et al., 2000 *x 2* [51] | Swales, 2010b [12]  Burroughs & Somerville, 2013 [15]  Swenson et al., 2002 *x 2* [21] |
|  | Facilitator skills | 2 | Chwalek & McKinney, 2015 *x 2* [24] |  |  |  |
|  | Staff selection | 32 | Ditty et al., 2015 *x 2* [26]  Cunningham et al., 2004 *x 5* [25]  Perseuis et al., 2007 [28]  Perseuis et al., 2003 [29]  Herschell et al., 2009 [36] | Ben-Porath et al., 2004 *x 2*  [53]  Brassington & Krawitz, 2006 [56]  Hjalmarsson et al., 2008 [59]  Pasieczny & Connor, 2011 [66]  Trupin et al., 2002 [69]  Van den Bosch et al., 2002 *x 3* [70]  Woodberry & Popenoe, 2008 [71] | Baillie & Slater, 2014 [39]  Lajoie et al., 2011 [44]  Little et al., 2010 [46] | Swales, 2010a *x 3* [11]  Burroughs & Somerville, 2013 [15]  Koerner, 2013 [18]  Swenson et al., 2002 *x 4* [21] |
|  | Team size | 18 | Carmel et al., 2014 [23]  Ditty et al., 2015 [26]  Fredrick & Comtois, 2006 [27]  Sharma et al., 2007 [30]  Herschell et al., 2009 [36] | Blennerhassett et al., 2009 *x 2* [54]  Hjalmarsson et al., 2008 [59]  Kerr et al., 2009 *x 2* [62]  Nee & Farman, 2005 [63]  Nelson-Gray et al., 2006 [64] | Morrissey & Ingamells, 2011 [47] | Swales, 2010a [11]  Berzins & Trestman, 2004 [14]  Burroughs & Somerville, 2013 [15]  Chugani, 2015 [16]  Scheel, 2000 [20] |
|  | Team configuration | 13 | Ditty et al., 2015 *x 2* [26]  Perseuis et al., 2007 [28] | Brassington & Krawitz, 2006 *x 2* [56]  Koons et al., 2006 *x 3* [65]  Pasieczny & Connor, 2011 [66] | Engle et al., 2013 [42]  Kinsey & Reed, 2015 [43]  Lajoie et al., 2011 [44]  Zinkler et al., 2007 [52] |  |
|  | Skills mix | 4 |  | Hjalmarsson et al., 2008 [59]  Van den Bosch et al., 2002 [70] | Little et al., 2010 [46] | Swales, 2010a [11] |
|  | Staff turnover | 19 | Carmel et al., 2014 [23]  Fredrick & Comtois, 2006 [27]  Perseuis et al., 2007 *x 2* [28]  Swales et al., 2012 *x 2* [31]  Herschell et al., 2014 [35]  Herschell et al., 2009 [36] | Ben-Porath et al., 2004 *x 3* [53]  Kerr et al., 2009 [62]  Nee & Farman, 2005 [63] | Little et al., 2010 [46]  Zinkler et al., 2007 [52] | Swales, 2010a [11]  Burroughs & Somerville, 2013 [15]  Swenson et al., 2002 *x 2* [21] |
| Training and ongoing support | | | | | | |
|  | Training | 65 | Carmel et al., 2014 *x 2* [23]  Chwalek & McKinney, 2015 *x 2* [24]  Fredrick & Comtois, 2006 *x 2* [27]  Perseuis et al., 2007 [28]  Sharma et al., 2007 *x 2* [30]  Swales et al., 2012 [31]  Dimeff et al., 2011 *x 2* [32]  Dimeff et al., 2009 *x 2* [33]  Hawkins & Sinha, 1998 *x 2* [34]  Herschell et al., 2014 *x 2* [35] | Blennerhassett et al., 2009 [54]  Brassington & Krawitz, 2006 [56]  Comtois et al., 2007 [57]  Hjalmarsson et al., 2008 *x 2* [59]  Katz et al., 2004 [61]  Kerr et al., 2009 [62]  Nee & Farman, 2005 *x 2* [63]  Nelson-Gray et al., 2006 [64]  Koons et al., 2006 [65]  Pasieczny & Connor, 2011 *x 2* [66]  Prendergast & McCausland, 2007 [67]  Rathus & Miller, 2002 [68]  Trupin et al., 2002 *x 3* [69]  Van den Bosch et al., 2002 *x 2* [70]  Woodberry & Popenoe, 2008 [71] | Barley et al., 1993 *x 2* [40]  Engle et al., 2013 [42]  Lajoie et al., 2011 [44]  Lew et al., 2006 [45]  Little et al., 2010 *x 2* [46]  Morrissey & Ingamells, 2011 *x 2* [47]  Sampl et al., 2010 [48]  Sunseri, 2004 *x 2* [49] | Swales, 2010a *x 2* [11]  Berzins & Trestman, 2004 [14]  Huffman et al., 2003 *x 2* [17]  Koerner, 2013 [18]  McHugh & Barlow, 2010 *x 2* [19]  Scheel, 2000 [20]  Swenson et al., 2002 *x 5* [21] |
|  | Coaching | 32 | Carmel et al., 2014 *x 2* [23]  Cunningham et al., 2004 [25]  Fredrick & Comtois, 2006 [27]  Perseuis et al., 2007 [28]  Dimeff et al., 2009 [33]  Hawkins & Sinha, 1998 [34]  Herschell et al., 2009 [36] | Katz et al., 2004 [61]  Kerr et al., 2009 [62]  Rathus & Miller, 2002 [68]  Trupin et al., 2002 [69]  Van den Bosch et al., 2002 *x 2* [70]  Woodberry & Popenoe, 2008 [71] | APA Gold Award, 2004 *x 2* [37]  Barley et al., 1993 [40]  Gold Award, 1998 [41]  Engle et al., 2013 [42]  Little et al., 2010 [46]  Sunseri, 2004 [49] | Swales, 2010a *x 4* [11]  Berzins & Trestman, 2004 [14]  Koerner, 2013 [18]  McHugh & Barlow, 2010 [19]  Scheel, 2000 [20]  Swenson et al., 2002 *x 2* [21] |
|  | Supervision | 27 | Cunningham et al., 2004 [25]  Ditty et al., 2015 [26]  Perseuis et al., 2007 *x 3* [28]  Sharma et al., 2007 *x 2* [30]  Swales et al., 2012 [31]  Dimeff et al., 2009 [33] | Ben-Porath et al., 2004 [53]  Hjalmarsson et al., 2008 *x 2* [59]  Katz et al., 2004 [61]  Kerr et al., 2009 *x 2* [62]  Nee & Farman, 2005 [63]  Prendergast & McCausland, 2007 [67]  Rathus & Miller, 2002 *x 2* [68]  Van den Bosch et al., 2002 [70] | Arroyo et al., 2012 [38]  Kinsey & Reed, 2015 [43]  Little et al., 2010 [46] | Swales, 2010a *x 2* [11]  Scheel, 2000 [20]  Swenson, 2000 [22] |
| ***DBT*** | | | | | | |
| Design quality and packaging | | | | | | |
|  | Design | 15 | Chwalek & McKinney, 2015 *x 2* [24]  Perseius et al., 2003 [29] | Blennerhassett et al., 2009 [54] | Little et al., 2010 [46]  Sunseri, 2004 *x 4* [49] | Berzins & Trestman, 2004 [14]  Huffman et al., 2003 [17]  Swenson et al., 2002 [21]  Swenson, 2000 *x 3* [22] |
|  | Manualisation | 10 | Chwalek & McKinney, 2015 [24]  Perseuis et al., 2003 [29]  Hawkins & Sinha, 1998 [34] | Van den Bosch et al., 2002 [70] | Morrissey & Ingamells, 2011 [47] | Berzins & Trestman, 2004 *x 2* [14]  Burroughs & Somerville, 2013 [15]  Koerner, 2013 [18]  Swenson et al., 2002 [21] |
|  | Complexity | 15 | Chwalek & McKinney, 2015 [24]  Cunningham et al., 2004 [25]  Swales et al., 2012 [31]  Hawkins & Sinha, 1998 [34] | Blennerhassett et al., 2009 [54]  Hjalmarsson et al., 2008 [59] | Lew et al., 2006 *x 2* [45]  Little et al., 2010 [46]  Morrissey & Ingamells, 2011 [47] | Koerner, 2013 [18]  Swenson et al., 2002 *x 2* [21]  Swenson, 2000 *x 2* [22] |
|  | Adaptations | 52 | Sharma et al., 2007 [30] | Ben-Porath et al., 2004 [53]  Bohus et al., 2000 [55]  Brassington & Krawitz, 2006 [56]  Comtois et al., 2007 [57]  Eccleston & Sobello, 2002 [58]  Hjalmarsson et al., 2008 [59]  James et al., 2015 [60]  Katz et al., 2004 [61]  Kerr et al., 2009 *x 2* [62]  Nee & Farman, 2005 *x 2* [63]  Nelson-Gray et al., 2006 [64]  Koons et al., 2006 *x 2* [65]  Prendergast & McCausland, 2007 [67]  Rathus & Miller, 2002 [68]  Trupin et al., 2002 *x 2* [69]  Van den Bosch et al., 2002 [70]  Woodberry & Popenoe, 2008 [71] | APA Gold Award, 2004 [37]  Arroyo et al., 2012 *x 2* [38]  Baillie & Slater, 2014 [39]  Barley et al., 1993 [40]  Gold Award, 1998 *x 2* [41]  Engle et al., 2013 [42]  Kinsey & Reed, 2015 *x 2* [43]  Lew et al., 2006 [45]  Little et al., 2010 [46]  Morrissey & Ingamells, 2011 [47]  Sampl et al., 2010 [48]  Sunseri, 2004 [49]  Vitacco & Van Rybroek, 2006 [50]  Wolpow et al., 2000 [51]  Zinkler et al., 2007 [52] | Berzins & Trestman, 2004 *x 9* [14]  Burroughs & Somerville, 2013 [15]  Chugani, 2015 *x 2* [16] |
| Cost | | | | | | |
|  | Cost | 36 | Fredrick & Comtois, 2006 [27]  Dimeff et al., 2011 [32]  Herschell et al., 2009 [36] | Ben-Porath et al., 2004 [53]  Blennerhassett et al., 2009 *x 2* [54]  Brassington & Krawitz, 2006 *x 2* [56]  James et al., 2015 [60]  Woodberry & Popenoe, 2008 [71] | APA Gold Award, 2004 *x 2* [37]  Baillie & Slater, 2014 [39]  Gold Award, 1998 *x 4* [41]  Lajoie et al., 2011 *x 2* [44]  Lew et al., 2006 [45]  Little et al., 2010 *x 2* [46]  Zinkler et al., 2007 *x 2* [52] | Berzins & Trestman, 2004 [14]  Burroughs & Somerville, 2013 *x 5* [15]  Chugani, 2015 *x 2* [16]  Swenson et al., 2002 *x 2* [21]  Swenson, 2000 *x 2* [22] |
| ***Implementation process*** | | | | | | |
| Implementation process | Implementation process | 23 | Carmel et al., 2014 *x 3* [23]  Swales et al., 2012 [31] | Blennerhassett et al., 2009 [54]  Brassington & Krawitz, 2006 [56]  Comtois et al., 2007 *x 2* [57]  Hjalmarsson et al., 2008 *x 2* [59]  Nee & Farman, 2005 *x 2* [63] | Barley et al., 1993 *x 2* [40]  Gold Award, 1998 *x 2* [41]  Morrissey & Ingamells, 2011 [47]  Sampl et al., 2010 [48]  Wolpow et al., 2000 [51]  Zinkler et al., 2007 [52] | Swales, 2010a *x 2* [11]  Swales, 2010b [12] |

**Key:** ^$^ Process analysis papers also constitute examples of local knowledge; ‘x *number*’ indicates number of times the code was assigned in the paper (if greater than once).

**Abbreviations:** BPD = Borderline Personality Disorder, DBT = Dialectical Behaviour Therapy
